# Entrepreneurial Team Flourishing Amidst AI Revolution: The Influence of AI Literacy on Hedonic and Eudaimonic Well-Being Through Efficacy and Anxiety

**DOI:** 10.3390/bs16071198

**Published:** 2026-07-16

**Authors:** Haiqing Hu, Yirong Liu, Weiwei Kong, Zhuoyi Li

**Affiliations:** School of Economics and Management, Xi’an University of Technology, Xi’an 710054, China; huhaiqing@xaut.edu.cn (H.H.); 1210512016@stu.xaut.edu.cn (W.K.); 1210510007@stu.xaut.edu.cn (Z.L.)

**Keywords:** AI literacy, entrepreneurial team well-being, entrepreneurial team efficacy, collective AI anxiety, conservation of resources

## Abstract

As artificial intelligence (AI) rapidly permeates entrepreneurial ecosystems, understanding how technological literacy relates to entrepreneurial team well-being has become an urgent priority. Drawing on conservation of resources (COR) theory, this study examines the relationship between entrepreneurial teams’ AI literacy and team well-being (distinguishing between hedonic and eudaimonic well-being). Furthermore, it investigates the mediating roles of entrepreneurial team efficacy and collective AI anxiety. Data were collected from a survey of 271 entrepreneurial teams across four major economic zones in China. This study relies on team leaders as primary informants to report team-level perceptions. The results show that AI literacy is positively related to team hedonic well-being, but exhibits no significant direct relationship with team eudaimonic well-being. However, AI literacy is indirectly associated with entrepreneurial team well-being through two parallel pathways: entrepreneurial team efficacy and collective AI anxiety. The findings shed light on how technological literacy is linked to team mental health via the dual mechanisms of cognitive resource gain and emotional loss prevention. This study broadens our understanding of well-being at the entrepreneurial team level and offers insights into cultivating literacy, enhancing efficacy, and managing emotions to improve entrepreneurial team well-being.

## 1. Introduction

The widespread adoption of AI has brought about increasingly profound changes across social domains, including operational, educational, and workplace settings. This technological wave is reshaping the entrepreneurial ecosystem, presenting entrepreneurs with novel opportunities yet unprecedented threats to their mental health (Giuggioli & Pellegrini, 2023; Valtonen et al., 2025). Against this backdrop, enhancing entrepreneurial well-being, which reflects perceptions and affective evaluations of venture experiences, becomes particularly important. Entrepreneurial well-being relates positively to resilience in high-pressure environments, willingness to innovate, opportunity assessment, and decision-making quality, thereby contributing to entrepreneurial success and performance (Stephan et al., 2023; Pak, 2026; Nikolaev et al., 2020). It is estimated that negative psychological states result in annual productivity losses exceeding one trillion U.S. dollars globally (Pinheiro et al., 2017). Currently, entrepreneurship is gradually shifting from individual-led efforts to team-based collaboration, with entrepreneurial teams exhibiting a higher success rate (Ye et al., 2021). However, deeply intertwined interests, loose structural norms, and fragile relational capital make entrepreneurial teams more vulnerable to intense emotional reactions and collective psychological depletion (Li et al., 2025; Feng et al., 2026). Despite this, existing research has largely focused on the well-being of individual entrepreneurs, overlooking the collective dimension at the entrepreneurial team level (Yu et al., 2025). This gap is particularly salient in the Chinese context, which emphasizes collectivism, interpersonal harmony, and shared well-being.

As AI becomes embedded in entrepreneurial practice, protecting team well-being from technological erosion while enabling its improvement has become an urgent priority. The rapid advancement of AI has significantly exacerbated major risks and psychological pressure, thereby increasing the demand for the knowledge and skills to understand and use AI (Huang & Zhao, 2025; Kaya et al., 2025). To address this, the present study extends AI literacy to the entrepreneurial team level. We operationalize this construct as the collective knowledge, attitudes, values, and ethical consensus regarding the understanding, evaluation, and application of AI technologies (Long & Magerko, 2020; Ng et al., 2021; Pinski et al., 2024). This form of literacy helps leverage AI to create value, fosters positive technological attitudes, and satisfies basic psychological needs (Huang & Zhao, 2025; Kaya et al., 2025). However, current entrepreneurship literature addressing AI literacy has primarily focused on its associations with entrepreneurial intention, orientation, and resilience (Duong, 2025; Kong et al., 2025; Polat et al., 2025; Liu et al., 2025). Although some studies have begun to explore the relationship between AI literacy and well-being, they are largely confined to educational or daily life contexts (Huang & Zhao, 2025; Alshowkan & Shdaifat, 2025; Shi et al., 2025; Kaya et al., 2025). Limited efforts have been directed toward the distinctive context of entrepreneurial teams, characterized by high uncertainty and intensive emotional interaction. Consequently, the field lacks an effective bridge linking entrepreneurial teams’ objective technical knowledge to their subjective well-being. This gap prevents static AI capabilities from translating into dynamic collective well-being in practice. In light of this, this paper focuses on AI literacy at the entrepreneurial team level and delves into its critical predictive role in entrepreneurial team well-being.

Based on COR theory, this study examines how entrepreneurial team AI literacy is associated with entrepreneurial team well-being through two parallel pathways: entrepreneurial team efficacy and collective AI anxiety. Although COR theory originated at the individual level, its logic extends naturally to teams as collective resource systems, where members jointly acquire, conserve, and protect resources (Monnens et al., 2026). From this perspective, entrepreneurial team AI literacy serves as a key shared technical resource. According to COR theory, teams with abundant initial resources tend to accumulate further gains or prevent losses to achieve future resource growth (Hobfoll, 2001). Entrepreneurial team efficacy, defined as members’ shared confidence in the team’s ability to successfully execute entrepreneurial tasks (Hmieleski & Cole, 2022), represents the cognitive path of resource gain. Collective AI anxiety, referring to shared concerns about the uncertainties and potential threats of AI (Nasaj et al., 2026), corresponds to the emotional path of resource loss prevention. These two paths, respectively from the dual dimensions of resource accumulation and threat mitigation, elucidate the internal mechanism through which AI literacy relates to entrepreneurial team well-being.

Although prior research offers valuable efforts, several gaps remain. Studies on entrepreneurial team efficacy mainly examine its consequences (Y. Chen et al., 2025; Schoss et al., 2022; Hmieleski & Cole, 2022), largely overlooking its antecedents. While some scholars advocate testing that examines efficacy as a mediator between technical literacy and well-being (Kaya et al., 2025), this pathway remains untested in entrepreneurial teams. Moreover, AI anxiety research has largely concentrated on the individual level, lacking exploration of such collective emotions. More critically, tensions exist in the current literature. Some studies report a negative association between high AI literacy and AI anxiety (Schiavo et al., 2024; Cengiz & Peker, 2025), whereas others identify a positive link between deeper AI knowledge and concerns (Canbeldek Erol et al., 2026). Regarding the consequences of AI anxiety, existing evidence suggests two contrasting findings. Some studies link it to diminished mental health and resilience (Soomro et al., 2026; Liu et al., 2025), while others suggest that it may be positively associated with team innovation (Nasaj et al., 2026). Thus, the precise role of collective AI anxiety in entrepreneurial teams requires further clarification.

**Figure 1:**
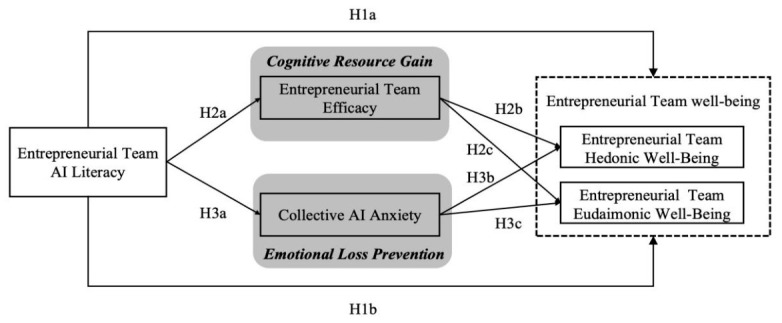
Theoretical model.

In summary, this study examines the impact of entrepreneurial team AI literacy on well-being and its underlying mechanisms. Drawing on COR theory, we test the dual mediating roles of entrepreneurial team efficacy and collective AI anxiety in the relationship between AI literacy and entrepreneurial team well-being. The findings extend COR theory to the nexus of entrepreneurial teams and AI technology, elucidating an integrated dual mechanism of cognitive resource gain and emotional loss prevention. At the practical level, this study offers targeted implications and actionable pathways for entrepreneurial teams to foster AI literacy, activate team agency, and manage technology-related emotions, thereby effectively enhancing overall well-being amidst technological change. The theoretical model is illustrated in Figure 1.

## 2. Literature Review and Hypothesis Development

### 2.1. Entrepreneurial Team Well-Being

In recent years, scholarly attention to well-being in the entrepreneurship domain has grown steadily. Well-being is critical not only at the individual level but also at the collective level (Anderson et al., 2013). In the entrepreneurial context, teams often operate in highly uncertain and ambiguous non-routine environments over extended periods (Li et al., 2025). Consequently, examining their overall psychological state and emotional experience becomes particularly critical. Yu et al. (2025) explicitly called for exploring the nature and antecedents of collective well-being at the entrepreneurial team level. Stephan et al. (2023) also noted the need for a more nuanced theoretical conceptualization of the meaning and components of well-being. While individual well-being reflects a person’s overall evaluation of their own life, collective well-being refers to the shared perception within a group (Rani et al., 2025). Entrepreneurial team well-being is neither merely the aggregate of individual members’ feelings nor benchmarked against a single member. Rather, it is conceptualized as a collective construct. Even though members experience different levels of individual well-being, the team may still develop a shared sense of its overall state. Accordingly, this study defines entrepreneurial team well-being as members’ collective experience of satisfaction, positive emotions, and optimal psychological functioning during the processes of venture preparation, establishment, growth, and operation (Wiklund et al., 2019; Ryan & Deci, 2000; Nikolaev et al., 2020).

The study of well-being is rooted in two distinct philosophical traditions, namely, hedonism and eudaimonism (Ryan & Deci, 2000). These traditions have informed a dual-dimensional framework that is also applicable at the collective level. However, existing studies have largely examined only one of these dimensions in isolation. To more comprehensively capture the complex nature of entrepreneurial team well-being, this study advocates incorporating both hedonic and eudaimonic dimensions into the analytical framework. Specifically, entrepreneurial team hedonic well-being refers to members’ positive evaluations of the team’s overall emotional experience and satisfaction, emphasizing affective states such as pleasure and contentment (Diener et al., 1999; Huta & Ryan, 2010). Entrepreneurial team eudaimonic well-being goes beyond a narrow and short-term view of psychological functioning, highlighting the team’s sustained flourishing through the pursuit of valuable goals, realization of collective potential, and acquisition of growth (Luu & Nguyen, 2024; Mukerjee et al., 2026). Building on this distinction, this study incorporates both hedonic and eudaimonic dimensions into a unified framework to examine how entrepreneurial team AI literacy relates to these two forms of well-being.

### 2.2. Entrepreneurial Team AI Literacy and Entrepreneurial Team Well-Being

The knowledge, skills, and values possessed by a team are critical factors influencing entrepreneurial adaptation and results (Guo et al., 2023). In a context where AI technology is deeply embedded in entrepreneurial practice, the team’s overall psychological state largely depends on its collective ability to understand, apply, and evaluate AI. COR theory suggests that people tend to actively seek, maintain, and protect valuable resources, which are associated with their well-being (Hobfoll et al., 2018). Accordingly, entrepreneurial team AI literacy is positively associated with team well-being by optimizing human–AI interaction, strengthening emotional bonds, and improving decision-making quality.

First, AI literacy equips the team with the ability to understand and effectively collaborate with AI technologies within ethical norms (B. Wang et al., 2023). This enables team members to more readily identify entrepreneurial value created by AI, thereby fostering positive attitudes toward technology adoption (Schiavo et al., 2024). Such positive attitudes may contribute to more harmonious human–AI relationships and greater task fluency, which are in turn associated with members’ satisfaction and positive emotional experiences (Kaya et al., 2025). Second, higher AI literacy facilitates smoother communication and more efficient information sharing within the team, thereby strengthening members’ sense of belonging and collective identity (Huang & Zhao, 2025). This helps create a cohesive emotional climate, which in turn is linked to greater team well-being. Third, greater AI literacy reduces the team’s over-reliance on subjective intuition, enabling data-driven, objective, and rational decision-making while mitigating cognitive and behavioral biases (Iram et al., 2026). This robust decision-making capacity allows the team to formulate and implement sound strategies more effectively, thereby generating a strong sense of goal attainment and collective meaning. In summary, we propose the following hypotheses:

**H1a.**  *Entrepreneurial team AI literacy is positively associated with entrepreneurial team hedonic well-being.*

**H1b.**  *Entrepreneurial team AI literacy is positively associated with entrepreneurial team eudaimonic well-being.*

### 2.3. The Mediating Role of Entrepreneurial Team Efficacy

COR theory suggests that people with abundant resources can continuously acquire new resources by investing those resources into tasks, thereby achieving resource gains (Hobfoll et al., 2018). Building on this, this study proposes that AI literacy, as an invested shared technical resource, is positively associated with team collective efficacy. Collective efficacy is a key social cognitive characteristic influencing team entrepreneurial processes and outcomes, and a core component of collective agency (Y. Chen et al., 2025). Teams with strong efficacy beliefs are confident in their ability to successfully execute entrepreneurial tasks, persist through setbacks, and recover quickly, thereby enhancing well-being (Drnovšek et al., 2026; Ribeiro et al., 2025).

On one hand, when teams possess high collective literacy in understanding AI, ethical judgment, and output evaluation, members can skillfully leverage this emerging technology (Pinski et al., 2024). Such mastery of AI significantly strengthens the team’s confidence in completing AI-related tasks, thereby increasing collective efficacy (Bachmann et al., 2024). Furthermore, high AI literacy helps teams integrate AI systems into specific entrepreneurial tasks such as opportunity recognition and decision-making, improving entrepreneurial success rates (Polat et al., 2025). The positive behavioral feedback further reinforces the team’s positive expectations of consistently performing various entrepreneurial tasks with the aid of AI.

On the other hand, high efficacy is associated with greater mutual dependence and recognition among team members, more open communication and collaboration, and a positive, trusting emotional climate (S. Chen et al., 2015). Such high-quality team interaction provides critical emotional support, enabling members to experience flow and satisfaction in their work, thereby sustaining positive affective states. Second, high team efficacy means that the team has greater confidence in coping with crises and withstanding shocks (Ribeiro et al., 2025). This belief motivates the team to boldly pursue challenging entrepreneurial goals and reinforces members’ sense of value and purpose in their teamwork (Yang et al., 2022; Y. Chen et al., 2025).

In summary, entrepreneurial team AI literacy is positively associated with stronger collective entrepreneurial efficacy, potentially reflected in strengthened technological mastery and accumulated successful experiences. Efficacy, in turn, is associated with greater emotional support and confidence in goal attainment, thereby linking to both hedonic and eudaimonic well-being. Based on the above reasoning, this study proposes the following hypotheses:

**H2.**  *Entrepreneurial team efficacy mediates the relationship between entrepreneurial team AI literacy and entrepreneurial team well-being.*

**H2a.**  *Entrepreneurial team AI literacy is positively related to entrepreneurial team efficacy.*

**H2b.**  *Entrepreneurial team efficacy is positively related to hedonic well-being.*

**H2c.**  *Entrepreneurial team efficacy is positively related to eudaimonic well-being.*

### 2.4. The Mediating Role of Collective AI Anxiety

According to COR theory, the resources people possess can help mitigate the impact of resource loss and may facilitate the accumulation of new resources (Hobfoll et al., 2018). In this study, entrepreneurial team AI literacy represents a protective shared technical resource linked to reduced psychological depletion stemming from technology-related uncertainty and risk concerns. The collective challenges and stress reactions associated with AI applications can be examined utilizing AI anxiety, which relates to the team’s overall psychological state (Salimi et al., 2025; Nasaj et al., 2026).

On one hand, AI literacy equips the team with the knowledge and ability to comprehensively understand, critically evaluate, and responsibly use AI (Qiu et al., 2025). This helps eliminate fear and worry caused by technological mystery or outcome uncertainty, thereby alleviating technology anxiety (Schiavo et al., 2024; Cengiz & Peker, 2025). Second, high AI literacy implies that team members possess the necessary knowledge base and have formed a collaborative technical support network within the team. This is associated with lower perceived difficulty in learning AI technologies and reduced collective helplessness related to skill acquisition challenges. Furthermore, a clear understanding of the boundaries of AI capabilities helps the team recognize the irreplaceable roles of human creativity and empathy, thereby reducing the perceived threat of being replaced by technology (Liu et al., 2025).

On the other hand, persistent concerns about AI substitution and anthropomorphism tend to trigger threat perceptions and stress, making the team’s psychological state more conservative and vigilant (Drnovšek et al., 2026). Conversely, reduced anxiety about being replaced or marginalized correlates with more open AI collaboration and increased pleasure among members. In addition, AI anxiety creates psychological and behavioral barriers to technology adoption (Zhang & Tong, 2025). Lower collective AI anxiety is associated with greater willingness to adopt AI and readiness for change (Suseno et al., 2022; Salimi et al., 2025). This helps the team successfully solve problems with AI and sustain improvements in collective productivity and goal attainment in practice. This, in turn, reinforces the sense of growth and value identification during the entrepreneurial process.

In summary, entrepreneurial team AI literacy significantly reduces collective AI anxiety by reducing technological opacity, lowering learning helplessness and weakening perceptions of substitution threats. Such technology anxiety is associated with resource depletion by triggering psychological tension and hindering technology adoption. This depletion may in turn diminish the team’s experience of pleasure and sense of achievement derived from entrepreneurial activities. Based on the above reasoning, this study proposes the following hypotheses:

**H3.**  *Collective AI anxiety mediates the relationship between entrepreneurial team AI literacy and entrepreneurial team well-being.*

**H3a.**  *Entrepreneurial team AI literacy is negatively related to collective AI anxiety.*

**H3b.**  *Collective AI anxiety is negatively related to hedonic well-being.*

**H3c.**  *Collective AI anxiety is negatively related to eudaimonic well-being.*

## 3. Methods

### 3.1. Participants and Procedures

The data for this study were collected from entrepreneurial teams in China. Leveraging our research group’s long-term cooperation with business incubators and entrepreneurship parks in Beijing, Wuhan, Xi’an, and Changchun, we conducted a questionnaire survey in these four cities. These four cities are located in the eastern, central, western, and northeastern economic regions of China as classified by the National Bureau of Statistics, and were selected as survey sites in order to ensure sample representativeness. With the assistance of park directors, we recruited eligible entrepreneurial teams. Eligibility was defined as currently being engaged in entrepreneurial activities with a team age of no more than 8 years. Participants were assured that all data would be anonymized and used exclusively for academic purposes without affecting themselves or their firms. All participants voluntarily joined the study after providing informed consent.

Before launching the formal survey, we conducted a pretest to validate the appropriateness of using primary informants for team-level measurement. Following the procedure of Hmieleski and Cole (2022), we first distributed questionnaires to primary informants (team leaders). After receiving valid responses from them, we then distributed questionnaires to secondary informants (other team members). A total of 28 teams provided complete data from both sources. To assess interrater agreement (IRA), we calculated the rWG(j) statistic, which indicates the absolute degree of consensus among respondents within the same team. The results showed that the mean rWG(j) values for all variables ranged from 0.81 to 0.92, exceeding the commonly accepted threshold of 0.70. The high consistency confirms that data provided by team leaders can effectively reflect team-level perceptions. Therefore, in the formal survey phase, we administered questionnaires only to primary informants. Primary informants in this study were team leaders who bore primary responsibility for strategic decision-making and operational management, such as CEOs. Respondents’ eligibility was double-checked through organizational structure information provided by park directors, as well as a self-identification item at the beginning of the questionnaire (“Are you the primary leader of this entrepreneurial team?”).

This study employed a time-lagged design with data collected in three waves. The first wave distributed paper questionnaires offline, while the subsequent two waves were conducted through an online survey platform (Credamo). The first wave was launched in October 2025, with an interval of 40 days between waves. Each participant was assigned a unique identification code for data tracking and matching across waves. In the first wave, participants assessed their team’s AI literacy level and provided demographic information. The second wave measured entrepreneurial team efficacy and collective AI anxiety. The third wave administered the entrepreneurial team well-being scale. A total of 340 questionnaires were distributed, and 301 were returned, yielding a response rate of 88.5%. We further adhered to exclusion criteria that were established before data analysis. Samples were excluded if they had completion times of less than 60 s or missing data rates exceeding 20%. This yielded a final valid sample of 271 questionnaires, representing an effective response rate of 79.7%.

In terms of firm age, 27.3% of the teams had been established for less than 2 years, the largest proportion (59.1%) had been operating for 2 to 4 years, and 13.6% for more than 4 years. Regarding team size, 32.8% of the teams had 2 to 5 members, 51.7% had 6 to 10 members, and 15.5% had 11 members or more. The industry distribution was mainly concentrated in internet and e-commerce, medical devices and health, manufacturing, and services.

### 3.2. Measures

Given that some of the measurement instruments were originally developed at the individual level, we followed a procedural adaptation to the team level. First, we shifted the referents of the items from individuals to teams and modified the wording to better align with the context. Second, three professors specializing in entrepreneurship management reviewed all adapted items. Based on expert feedback, we further optimized the item phrasing to ensure the appropriateness of team-level wording while preserving the original conceptual connotations. Finally, a pretest was conducted to examine the suitability of all scale items. Since the original scales were in English, we implemented a translation and back-translation procedure under the guidance and supervision of these three professors to ensure conceptual equivalence. Participants were asked to rate each item based on the overall situation of their team. All constructs were measured using a five-point Likert scale ranging from 1 (strongly disagree) to 5 (strongly agree).

Entrepreneurial team well-being was conceptualized as consisting of two dimensions: hedonic well-being and eudaimonic well-being. Hedonic well-being was assessed using five items adapted from Duan et al. (2025), with the referent shifted to the team level (e.g., Entrepreneurial team members are satisfied with their present state of life and work). The Cronbach’s α for this scale was 0.897. Eudaimonic well-being was measured by adapting Ryff’s (1989) multidimensional well-being scale. The original scale comprises six dimensions—autonomy, environmental mastery, personal growth, positive relations, self-acceptance, and purpose in life—with a total of 42 items. We adopted a concise measurement approach by selecting the single most representative item from each dimension. Specifically, we first reviewed the original item pool and identified the items that best captured the core essence of each dimension and were most relevant to our research context. These candidate items were then evaluated by three professors specializing in entrepreneurship management. They assessed each item based on its representativeness, clarity, and suitability for the team context. Only items that received unanimous approval from all three professors were retained. As the original scale was developed at the individual level, we shifted the referents from individuals to the team and further refined the wording under the guidance of three professors. An example item is: entrepreneurial team members have goals and a clear sense of direction in their venture. The scale demonstrated good internal consistency (Cronbach’s α = 0.904).

Entrepreneurial team AI literacy was measured using an adapted scale that integrated dimensions from Imjai et al. (2025) and B. Wang et al. (2023). Imjai et al. (2025) focused on four dimensions, emphasizing basic AI cognition, application proficiency, insight thinking, and analytical skills. In addition, B. Wang et al. (2023) highlighted the critical role of AI ethics in the construct of AI literacy to ensure that technological practices align with ethical norms and social responsibility. Integrating the above literature, this study developed a measurement framework covering five dimensions: AI Basics, AI proficiency, AI insight, and AI analysis and AI ethics, with a total of 11 items. To fit the team-level analysis of this study, we shifted the referent from the individual to the team, requiring participants to rate based on the overall AI literacy level of their team members. The overall scale showed excellent reliability (Cronbach’s α = 0.912).

Entrepreneurial team efficacy was measured using the scale adapted from Hmieleski and Cole (2022). This scale assesses the team’s collective confidence in six key entrepreneurial capabilities: developing new products and market opportunities, fostering an innovative environment, building investor relationships, clarifying core goals, responding to unexpected challenges, and developing core human resources. All items used a five-point Likert scale, with higher scores indicating stronger collective efficacy beliefs regarding the team’s overall entrepreneurial competence. The Cronbach’s α for this scale was 0.891.

Collective AI anxiety was measured using the team version adapted by Nasaj et al. (2026) based on the original AI anxiety scale developed by Y. Y. Wang and Wang (2022). Participants were asked to rate based on the team’s collective emotional state. The scale covers four dimensions: learning, job replacement, sociotechnical blindness, and AI configuration, with a total of 21 items (Cronbach’s α = 0.926).

The following control variables were included to adjust for potential confounding. First, team entrepreneurial experience was measured by team age (years since establishment), reflecting the team’s accumulated collective knowledge and coping ability in the entrepreneurial process (Marzi & Balzano, 2025). Second, team size was measured by the total number of team members. Team size has been linked to team processes and outcomes and is a commonly used control variable in team-level research (Monnens et al., 2026). In addition, to control for potential industry effects, we followed the approach of Hmieleski and Cole (2022) by coding industry categories and creating three dummy variables: retail, wholesale and services, manufacturing, and others.

### 3.3. Statistical Analyses

Statistical analyses were performed using SPSS 27.0 and AMOS 24.0. First, confirmatory factor analysis (CFA) was conducted to assess model fit using indicators such as χ^2^/df, CFI, TLI, RMSEA, and SRMR. Second, Cronbach’s α, composite reliability (CR), and average variance extracted (AVE) were used to evaluate the reliability and convergent validity of the scales. Discriminant validity was tested by comparing the square root of the AVE with the correlations among variables. Finally, structural equation modeling (SEM) was constructed using AMOS 24.0 to test the proposed hypotheses holistically. SEM has the advantage of simultaneously handling complex relationships among multiple variables and effectively examining mediating pathways.

## 4. Results

### 4.1. Descriptive Statistics

Descriptive statistics and correlations are summarized in Table 1. Entrepreneurial team AI literacy exhibited positive correlations with entrepreneurial team efficacy (r = 0.431, *p* < 0.001), hedonic well-being (r = 0.412, *p* < 0.001), and eudaimonic well-being (r = 0.389, *p* < 0.001), while showing a negative correlation with collective AI anxiety (r = −0.306, *p* < 0.001). These findings offer initial empirical support.

### 4.2. Measurement Validation

We first conducted a confirmatory factor analysis (CFA). The results indicated a good fit, as reflected by the following fit indices: χ^2^/df = 2.163, CFI = 0.958, TLI = 0.954, RMSEA = 0.062, SRMR = 0.042. Reliability and validity results are shown in Table 2. Standardized factor loadings of all measurement items exceeded 0.7, average variance extracted (AVE) values were above 0.5, and composite reliability (CR) ranged from 0.892 to 0.927, all exceeding recommended thresholds. Moreover, the square root of the AVE for each latent variable was greater than the correlations between that variable and other latent variables, demonstrating good discriminant validity. In summary, the measurement model achieved desirable levels of reliability, convergent validity, and discriminant validity.

### 4.3. Common Method Bias

To address common method bias (CMB), this study implemented several procedural and statistical measures. In the data collection process, we adopted a three-wave time-lagged design, guaranteed participants full anonymity, and randomized the order of questionnaire items. To further test CMB, we conducted both Harman’s single-factor test and a common latent factor (CLF) test. Harman’s test showed that the first unrotated factor accounted for 27.19% of the total variance, below the recommended threshold of 40%. The CLF test further indicated that the changes in standardized regression coefficients for all variables remained within 0.2 after the inclusion of the CLF. These findings suggest that common method bias does not pose a serious threat to the validity of our results.

### 4.4. Hypothesis Testing Results

The structural model exhibited a satisfactory fit to the data, with χ^2^/df = 2.215, CFI = 0.951, TLI = 0.948, RMSEA = 0.065, and SRMR = 0.048. Table 3 summarizes the hypothesis testing results. Entrepreneurial team AI literacy was positively associated with hedonic well-being (*β* = 0.223, *p* < 0.001), supporting H1a. However, the direct association between entrepreneurial team AI literacy and eudaimonic well-being was not significant. Thus, H1b was not supported. Furthermore, the analysis showed that entrepreneurial team AI literacy was significantly positively related to entrepreneurial team efficacy (*β* = 0.431, *p* < 0.001) and significantly negatively related to collective AI anxiety (*β* = −0.318, *p* < 0.001). In addition, entrepreneurial team efficacy positively predicted hedonic well-being (*β* = 0.198, *p* < 0.01) and eudaimonic well-being (*β* = 0.517, *p* < 0.001). Collective AI anxiety was negatively associated with hedonic well-being (*β* = −0.497, *p* < 0.001) and also demonstrated a significant negative association with eudaimonic well-being (*β* = −0.187, *p* < 0.01).

To test the mediating effects, we conducted path analysis using the bootstrap method with 5000 resamples (see Table 4). The results revealed that entrepreneurial team efficacy and collective AI anxiety serve as significant mediators in the relationship between AI literacy and hedonic well-being. The indirect effect via entrepreneurial team efficacy had an effect size of 0.085 with a 95% confidence interval that did not include zero [0.026, 0.156]. The indirect effect via collective AI anxiety had an effect size of 0.158 with a 95% confidence interval [0.083, 0.244]. Both mediating pathways were also significant in the relationship between AI literacy and eudaimonic well-being. Thus, H2 and H3 were supported, indicating that entrepreneurial team efficacy and collective AI anxiety both serve as significant mediating mechanisms in linking entrepreneurial team AI literacy to both types of well-being. Furthermore, we conducted pairwise comparisons of the indirect effects to examine differences in pathway strength. The results indicated that in the relationship between AI literacy and hedonic well-being, the indirect pathway through collective AI anxiety was larger than the pathway through entrepreneurial team efficacy (difference = 0.073, 95% CI [0.016, 0.141]). Conversely, for eudaimonic well-being, the pathway via entrepreneurial team efficacy was significantly larger than that via collective AI anxiety (difference = 0.164, 95% CI [0.076, 0.259]). These findings provide robust empirical evidence for the differentiated roles of cognitive resource gain and emotional loss prevention mechanisms across distinct well-being dimensions.

## 5. Discussion

This study integrates the literature on technical literacy, efficacy, anxiety, and well-being to establish theoretical linkages among these constructs. Using COR theory as the core analytical framework, we examined the effects of entrepreneurial team AI literacy on both hedonic and eudaimonic team well-being, and further tested the parallel mediating roles of entrepreneurial team efficacy and collective AI anxiety. The results reveal dimensional differences in the effects of AI literacy on entrepreneurial team well-being. Specifically, AI literacy has a significant direct positive effect on hedonic well-being, but its direct effect on eudaimonic well-being is not significant. Moreover, the indirect association between AI literacy and team well-being operates through two parallel pathways: entrepreneurial team efficacy (cognitive resource gain) and collective AI anxiety (emotional loss prevention). These two pathways center on resource accumulation and threat mitigation, respectively. Together, they illuminate the internal logic through which technical literacy relates to team well-being via dual mechanisms. At the same time, these findings extend the explanatory boundaries of COR theory in AI-driven entrepreneurship. These findings provide a new theoretical perspective for understanding entrepreneurial team well-being and offer practical evidence for the management and flow of team psychological resources in the AI era.

### 5.1. Theoretical Implication

First, this study extends the scope of entrepreneurial well-being research from the individual to the team level, expanding the explanatory boundaries of well-being theory in the entrepreneurship domain. Compared with the extensive literature focusing on individual entrepreneurs’ well-being, entrepreneurial team well-being has remained underexplored. To address this, we extend the analysis of well-being to the team level, capturing it through the perceptions of team leaders as primary informants. In doing so, this study clarifies the conceptual meaning of entrepreneurial team well-being and identifies its key antecedents in the AI era. This effort responds to the call for in-depth exploration of entrepreneurial well-being and its influencing factors at the team level (Yu et al., 2025), filling a gap in the literature regarding the collective psychological state of entrepreneurial teams. This understanding resonates with the traditional Chinese philosophy that “dú yuè lè bù rú zhòng yuè lè” (sharing happiness with others makes it more enjoyable), reflecting values that emphasize interpersonal harmony and shared well-being. Furthermore, this study enriches the theoretical dimensions of entrepreneurial well-being. Prior research has tended to focus either on satisfaction experiences or on feelings of growth and meaning, rarely integrating both into a unified framework. By incorporating both hedonic and eudaimonic dimensions, this study offers a more integrated theoretical perspective for understanding entrepreneurial team well-being. This dual-dimensional framework highlights that the psychological value of collective entrepreneurial activities lies not only in the immediate experience of positive emotions but also in deeper psychological processes such as collective goal attainment, potential realization, and meaning construction.

Second, this study examines the role of AI literacy in the entrepreneurial team context and reveals its differentiated associations with entrepreneurial team well-being. By conceptualizing AI literacy as a collective technical resource, we extend its research context to the entrepreneurial team. This enables the entire entrepreneurial team to develop a common language on AI-related issues, offering a new perspective on the core competencies required for entrepreneurial teams in the AI era. Moreover, existing entrepreneurship literature has primarily focused on the relationships between AI literacy and entrepreneurial intention, orientation, and resilience (Duong, 2025; Kong et al., 2025; Polat et al., 2025; Liu et al., 2025). However, little is known about the relationship between AI capabilities and dynamic collective well-being in entrepreneurial practice. This study bridges this gap by validating the mechanism linking AI literacy to entrepreneurial well-being, thereby extending the theoretical boundaries of research on antecedents of entrepreneurial team well-being.

A noteworthy finding is that although entrepreneurial team AI literacy is positively and directly associated with hedonic well-being, its direct association with eudaimonic well-being is not significant. While prior studies have generally documented a positive relationship between AI literacy and well-being (Huang & Zhao, 2025; Shi et al., 2025; Kaya et al., 2025), these conclusions were largely drawn from educational or everyday life contexts without differentiating between well-being dimensions. Our findings suggest that in the entrepreneurial context, which is characterized by high uncertainty and intense pressure, the psychological returns of technical literacy may be inherently more complex. One plausible explanation is that AI literacy, as a technical resource, offers immediate practical value. These immediate rewards directly contribute to team hedonic well-being by providing instant gratification and positive emotional experiences. In this sense, AI literacy functions as a pragmatic tool capable of delivering rapid returns. Eudaimonic well-being, however, pertains to deeper and more enduring facets of team functioning, namely, collective goal attainment, meaning construction, and purposeful direction. These outcomes are unlikely to be achieved solely through the possession or use of technical resources. Rather, they require time for teams to integrate AI capabilities into strategic thinking, align them with long-term vision, and navigate associated uncertainties and complexities. While AI literacy provides the necessary know-how, translating this know-how into a profound sense of shared meaning, growth, and purpose may necessitate additional enabling conditions.

Third, drawing on COR theory, this study reveals the dual mediating roles of entrepreneurial team efficacy and collective AI anxiety in the relationship between AI literacy and well-being. In doing so, it validates the operating mechanisms of resource gain and resource loss prevention motives in the AI-driven entrepreneurial context. AI literacy functions as an invested resource in the efficacy pathway, promoting well-being by enhancing the team’s confidence in its own capabilities, reflecting the logic of cognitive empowerment. In the anxiety pathway, it serves as a protective resource, safeguarding well-being by buffering technology-related uncertainty and threat perceptions, highlighting the role of emotional buffering. This finding supports the theoretical view that resource functions should match their coping nature (Mukerjee et al., 2026), and also resonates with the universal human tendency to seek benefits and avoid harms. These results are consistent with prior work linking AI literacy to higher efficacy and lower AI anxiety (Schiavo et al., 2024; Cengiz & Peker, 2025) and extend these associations to the team level. Moreover, collective AI anxiety has been positively associated with innovation in some studies (Nasaj et al., 2026). In the entrepreneurial team context of this study, however, it is negatively associated with psychological outcomes. A more nuanced finding is that the two mediating pathways differ in their associations with distinct well-being dimensions. The mediating role of entrepreneurial team efficacy is stronger in the relationship between AI literacy and eudaimonic well-being, whereas the mediating role of collective AI anxiety is stronger in the relationship between AI literacy and hedonic well-being. This may suggest that eudaimonic well-being depends more on the growth and meaning derived from efficacy enhancement, while hedonic well-being is more sensitive to emotional states and stress buffering. These findings highlight the critical bridging role of cognitive and emotional mediating mechanisms in the transformation of technical capabilities into psychological experiences. They offer nuanced insights for understanding the management of team psychological resources in the AI era.

### 5.2. Practical Implications

The findings of this study offer the following practical implications. First, AI literacy may serve as a key direction for team capability building. This study found a significant positive association between entrepreneurial team AI literacy and collective well-being. Such literacy extends beyond tool-level operations to encompass technical understanding, applied analytics, and ethical judgment. Based on this, incubators, industry associations, and relevant support organizations could attempt to explore the integration of modular AI literacy programs into entrepreneurship education and training systems. For instance, a series of AI literacy workshops could be designed with a three-module structure. The first module focuses on technical proficiency to ensure that teams acquire foundational skills in AI tool application. The second module emphasizes value assessment, guiding members to critically evaluate the appropriate boundaries and potential risks of AI use. The third module delves into ethical deliberation, assisting teams in collectively establishing internal consensus on technology ethics norms.

Second, this study identified entrepreneurial team efficacy and collective AI anxiety as two mediating pathways through which AI literacy is associated with team well-being. Building on these findings, two potential directions may be explored to enhance entrepreneurial team well-being. Drawing on the logic of cognitive resource gain underlying the efficacy pathway, teams may consider breaking down overall goals into actionable AI-executable short-term tasks and supplementing these efforts with shared success journals. Drawing on the logic of emotional loss prevention underlying the anxiety pathway, teams may consider holding AI emotion roundtables to openly discuss technology-related concerns and to mutually acknowledge both individual and collective efforts and resilience throughout the adaptation process.

Third, hedonic and eudaimonic well-being are empirically distinguishable and exhibit different patterns of association with the above mediating pathways. This suggests that team leaders may consider adopting a diagnostic approach when seeking to support team well-being. This involves tailoring strategies to the team’s current psychological needs and developmental stage. Specifically, when a team experiences low morale or declining satisfaction, priority may be given to mitigating AI-related anxiety. Conversely, when teams face existential challenges such as a loss of direction or insufficient growth momentum, the focus should shift toward reinforcing collective efficacy. These contextualized strategy prioritizations may enable more precise and effective well-being interventions.

### 5.3. Limitations and Future Directions

This study has several limitations, which also point to fruitful directions for future research. First, although the time-lagged design helps to reduce concerns about simultaneity and common method bias, causal interpretations should still be made with caution. Future research could adopt longitudinal tracking or intervention designs to dynamically capture the trajectories of AI literacy, entrepreneurial team efficacy, collective AI anxiety, and well-being over time, thereby providing stronger evidence for causal relationships. Second, this study relied solely on team leaders as primary informants, with all variables measured through self-report questionnaires. Although the responses of primary informants were highly consistent with those of other team members as demonstrated in the pretest, perceptions based on a single informant may still be subject to individual traits, cognitive biases, and subjective evaluative tendencies. Future research could consider collecting multi-member data or incorporating objective behavioral indicators to more reliably capture and validate shared team states.

Third, this study was conducted within the Chinese context, and whether the conclusions can be extended to other countries or cultural settings remains to be examined. Entrepreneurial environments, AI policies, technological infrastructure, and cultural values (e.g., individualism vs. collectivism) may differ substantially across countries. Future research could extend the model to diverse national and cultural contexts to examine the cross-cultural stability of these relationships. Finally, this study did not account for boundary conditions concerning the observed relationships. Future research could incorporate organizational culture, team mindfulness, leadership style, or external support as moderators to further clarify the contextual mechanisms through which AI literacy relates to well-being via efficacy and anxiety.

## 6. Conclusions

Drawing on COR theory, this study examines the relationship between entrepreneurial team AI literacy and entrepreneurial team well-being and explores its underlying mechanisms. The findings reveal that entrepreneurial team AI literacy is positively associated with hedonic well-being, whereas its direct association with eudaimonic well-being is not significant. Both entrepreneurial team efficacy and collective AI anxiety serve as mediators, representing the cognitive resource gain and the emotional loss prevention pathway, respectively. These findings extend the research horizon of entrepreneurial well-being to the team level and offer important practical implications for enhancing entrepreneurial team psychological flourishing in the AI era through cultivating literacy, enhancing efficacy, and managing emotions.

## Figures and Tables

**Table 1:** Descriptive statistics and correlations.

Variable	Mean	SD	1	2	3	4	5
1. Entrepreneurial team AI literacy	3.452	0.812	1				
2. Entrepreneurial Team Efficacy	3.798	0.664	0.431 ***	1			
3. Collective AI Anxiety	2.945	0.865	−0.306 ***	−0.274 **	1		
4. Entrepreneurial Team Hedonic Well-Being	3.652	0.746	0.412 ***	0.356 ***	−0.476 ***	1	
5. Entrepreneurial Team Eudaimonic Well-Being	3.580	0.771	0.389 ***	0.482 ***	−0.391 **	0.573 ***	1

Notes: ** *p* < 0.01; *** *p* < 0.001.

**Table 2:** Measurement Model.

Variables	Items	Factor Loadings	CR	AVE
Entrepreneurial Team Artificial Intelligence Literacy	ETAIL1	0.747	0.915	0.702
	ETAIL2	0.915		
	ETAIL3	0.783		
	ETAIL4	0.811		
	ETAIL5	0.792		
	ETAIL6	0.821		
	ETAIL7	0.845		
	ETAIL8	0.829		
	ETAIL9	0.788		
	ETAIL10	0.906		
	ETAIL11	0.894		
Entrepreneurial Team Efficacy	ETE1	0.768	0.892	0.665
	ETE2	0.812		
	ETE3	0.795		
	ETE4	0.843		
	ETE5	0.826		
	ETE6	0.797		
Collective Artificial Intelligence Anxiety	CAIA1	0.851	0.927	0.743
	CAIA2	0.884		
	CAIA3	0.836		
	CAIA4	0.805		
	CAIA5	0.891		
	CAIA6	0.868		
	CAIA7	0.913		
	CAIA8	0.824		
	CAIA9	0.903		
	CAIA10	0.817		
	CAIA11	0.882		
	CAIA12	0.826		
	CAIA13	0.845		
	CAIA14	0.912		
	CAIA15	0.791		
	CAIA16	0.823		
	CAIA17	0.782		
	CAIA18	0.853		
	CAIA19	0.864		
	CAIA20	0.915		
	CAIA21	0.875		
Entrepreneurial Team Hedonic Well-being	ETHWB1	0.815	0.899	0.638
	ETHWB2	0.832		
	ETHWB3	0.860		
	ETHWB4	0.791		
	ETHWB5	0.743		
Entrepreneurial Team Eudaimonic Well-being	ETEWB1	0.816	0.905	0.622
	ETEWB2	0.782		
	ETEWB3	0.841		
	ETEWB4	0.795		
	ETEWB5	0.853		
	ETEWB6	0.732		

**Table 3:** Direct effects of each model path.

Direct Effect	β	SE
Entrepreneurial Team AI Literacy → Entrepreneurial Team Hedonic Well-Being	0.223 ***	0.048
Entrepreneurial Team AI Literacy → Entrepreneurial Team Eudaimonic Well-Being	0.131	0.064
Entrepreneurial Team AI Literacy → Entrepreneurial Team Efficacy	0.431 ***	0.068
Entrepreneurial Team AI Literacy → Collective AI Anxiety	−0.318 ***	0.059
Entrepreneurial Team Efficacy → Entrepreneurial Team Hedonic Well-Being	0.198 **	0.067
Entrepreneurial Team Efficacy → Entrepreneurial Team Eudaimonic Well-Being	0.517 ***	0.074
Collective AI Anxiety → Entrepreneurial Team Hedonic Well-Being	−0.497 ***	0.071
Collective AI Anxiety → Entrepreneurial Team Eudaimonic Well-Being	−0.187 **	0.063

Notes: ** *p* < 0.01; *** *p* < 0.001.

**Table 4:** Indirect effects result (bootstrapping, *N* = 5000).

Indirect Effect	Effect	95% CI
Entrepreneurial Team AI Literacy → Entrepreneurial Team Efficacy → Entrepreneurial Team Hedonic Well-Being	0.085	[0.026, 0.156]
Entrepreneurial Team AI Literacy → Entrepreneurial Team Efficacy → Entrepreneurial Team Eudaimonic Well-Being	0.223	[0.134, 0.319]
Entrepreneurial Team AI Literacy → Collective AI Anxiety → Entrepreneurial Team Hedonic Well-Being	0.158	[0.083, 0.244]
Entrepreneurial Team AI Literacy → Collective AI Anxiety → Entrepreneurial Team Eudaimonic Well-Being	0.059	[0.011, 0.122]

## Data Availability

The raw data supporting the conclusions of this article will be made available by the authors upon reasonable request.

## Abbreviations

The following abbreviations are used in this manuscript:

AI	Artificial intelligence
CAIA	Collective Artificial Intelligence Anxiety
COR	Conservation of Resources
ETAIL	Entrepreneurial Team Artificial Intelligence Literacy
ETE	Entrepreneurial Team Efficacy
ETEWB	Entrepreneurial Team Eudaimonic Well-being
ETHWB	Entrepreneurial Team Hedonic Well-being
